# AI in Pediatric Spine Care: Clinical, Research, and Ethical Considerations

**DOI:** 10.3390/jcm14228115

**Published:** 2025-11-16

**Authors:** Hans K. Nugraha, Adam P. Rasmussen, Kellen L. Mulford, Linjun Yang, Cody C. Wyles, A. Noelle Larson

**Affiliations:** 1 Department of Orthopedic Surgery, Orlando Health, Orlando, FL 32806, USA; hans.nugraha@orlandohealth.com; 2 Department of Orthopedic Surgery, Mayo Clinic, Rochester, MN 55905, USA; rasmussen.adam@mayo.edu (A.P.R.); wyles.cody@mayo.edu (C.C.W.); 3 Orthopedic Surgery Artificial Intelligence Laboratory, Rochester, MN 55905, USA; mulford.kellen@mayo.edu (K.L.M.); yang.linjun@mayo.edu (L.Y.)

**Keywords:** artificial intelligence, pediatric spine, machine learning, large language modeling

## Abstract

Artificial intelligence (AI) is increasingly shaping pediatric spine care, leveraging its rapid advancements in healthcare to improve efficiency, accuracy, and disease understanding. Moreover, machine learning and deep learning excel at detecting complex patterns. This holds promise in processing spinal deformity data, with the potential to surpass traditional statistical methods in predictive accuracy. Challenges persist, however, including unclear clinical implementation guidelines, limited model transparency, and ethical concerns surrounding data privacy and bias. Small sample sizes and the need for larger, diverse datasets further complicate integration. In order to realize AI’s transformative potential in pediatric spine care, these critical obstacles must be addressed for effective and ethical clinical adoption. This review examines the role of AI through applications such as image sorting, surgical outcome prediction, forecasting of spinal curve progression, and vertebral volumetric analysis using deep reasoning. It also explores possible intraoperative contributions from AI, including robotics and optimized screw trajectory planning, and the potential of large language models in clinical practice.

## 1. Introduction

Over the past two decades, electronic health record (EHR) adoption has surged in developed countries. In the U.S., this was driven by political support, financial incentives, and the structured, meaningful use program [1]. However, when compared to countries that have not yet adopted EHRs, healthcare performance often falls short in enhancing patient care and expense, healthcare delivery, and stakeholder value [2]. Centralized data and imaging repositories, however, provide opportunities for large-scale data analytics, including those in pediatric spine care.

Artificial intelligence (AI) has progressed rapidly in everyday applications and is now increasingly being developed for clinical use in healthcare. It holds great promise for improving efficiency, accuracy, and disease understanding by recognizing both known and novel patterns in complex medical data. This includes applications in categorical image classification of large registries of radiographic images [3] and predicting surgical outcomes [4], tasks that traditionally require time-consuming and costly analysis by trained professionals.

**Figure 1:**
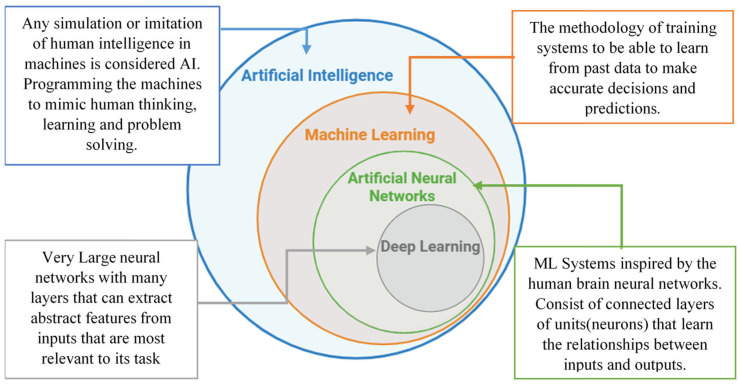
Artificial intelligence and its associated subsets. AI = artificial intelligence, ML = machine learning.

Through techniques from the fields of machine learning (ML) and deep learning (DL) (Figure 1), AI systems can detect complex, nonlinear relationships between data and outcomes. This is particularly useful in the presence of nonlinear relationships in spinal deformity data, such as how patient satisfaction relates to health-related quality of life (HRQoL) and complications [5]. While animal models offer valuable insights into the biomechanical effects of degenerative spinal conditions, machine learning has proven to be a more effective tool for predicting curve progression in complex developmental spinal deformities such as adolescent idiopathic scoliosis (AIS) [6].

Nevertheless, when it comes to ML models, the absence of clear clinical implementation guidelines in managing pediatric spinal deformity, the lack of model transparency, and external validation still hinders clinician trust and limits the generalizability and practical application of AI [7]. Additionally, there are challenges associated with patients’ individual variability, such as growth potential [8,9], small sample sets [10], and ethical concerns [11] in using AI in pediatric spine care. This review addresses a critical gap by synthesizing current AI applications in pediatric spine care, evaluating barriers to clinical translation, and proposing pathways for safe, effective integration. We focus on diagnostic, prognostic, and intraoperative uses of machine learning and deep learning, with emphasis on model interpretability, validation, and pediatric-specific ethical considerations.

## 2. Limitations of Traditional Statistics

Traditional statistical approaches, such as *t*-tests and analysis of variance, have been historically used to develop models using simple, sometimes univariate, inputs and outputs. These methods typically aim to achieve both inference (understanding why and how relationships exist) and prediction (forecasting what will happen). To provide valid inference, traditional statistical methods must be interpretable and cannot function as black-box models, which can sometimes limit their predictive performance. This top–down method assumes a direct link between independent and dependent variables that is often linear and may ignore other important, subtle contributing factors [12]. While traditional statistical methods, particularly simpler ones like *t*-tests or analysis of variance, commonly used in pediatric spine care, struggle with high-dimensional data, nonlinear relationships, or complex variable interactions, more advanced nonparametric statistical methods, such as regression splines, smoothing splines, or kernel smoothing, can address these issues. These methods, developed long ago and supported by classical statistical theory, are considered traditional but are less commonly used in medical studies like pediatric spine care due to their complexity and lower interpretability. Consequently, the limitations discussed here primarily reflect those of the simpler statistical methods most prevalent in this field.

Conversely, ML uses a bottom–up approach, beginning with a pool of data and developing a complex model from many variables. ML methods primarily focus on prediction, often prioritizing accuracy over interpretability, allowing for more complex models that may function as opaque “black-box” models. Explainable AI techniques, such as SHAP (SHapley Additive exPlanations) values or feature importance rankings, can be applied post hoc to enhance interpretability without sacrificing predictive performance. The resulting model has complex parameters that can be difficult to interpret. While it can result in more accurate predictions, ML requires much larger datasets than traditional statistics to tune its predictions accurately [13]. Thus, ML methods are preferred when prediction is the primary goal, while traditional statistical methods are advantageous for inference due to their interpretability.

Both traditional statistical techniques and ML can be used to predict continuous outcomes, such as the degree of scoliosis progression. For the purposes of illustration, we will walk through an example of both approaches to the prediction of scoliosis progression in an adolescent patient. Two risk factors (initial spinal curvature and age at diagnosis) will be used to predict or describe the degree of scoliosis progression. In the following paragraphs, it is described how multiple regression (traditional statistics) and Random Forest regression (ML technique) can tackle this problem.

In multiple regression, a model equation is created where the degree of scoliosis progress is described by the inputs via a mathematical function. The baseline risk corresponding to minimal spinal curvature and younger age at diagnosis is defined by a specific parameter. If there is some additional variable (for example, family history or initial spinal flexibility), this can be added to the model as well.

This method is relatively straightforward in the case of only two to three risk factors to consider. However, a more realistic scenario is one with a larger number of possible contributing predictors, such as sex, growth rate, skeletal maturity, spinal flexibility, physical activity levels, BMI, bracing compliance, and patient genetics and epigenetics. In that case, the situation may quickly become extremely complex. All possible pairs of predictors and their potential interactions (and maybe even nonlinear effect types) must be considered, making it difficult to detect and quantify individual contributions given the magnitude of the equation. While advanced nonparametric statistical methods could model such complexities, their use in pediatric spine care is limited due to interpretability challenges. The advantage of multiple regression lies in the fact that, once the model is defined, the process of describing scoliosis progression risk for each new patient is straightforward, easy to understand, and reproducible.

**Figure 2:**
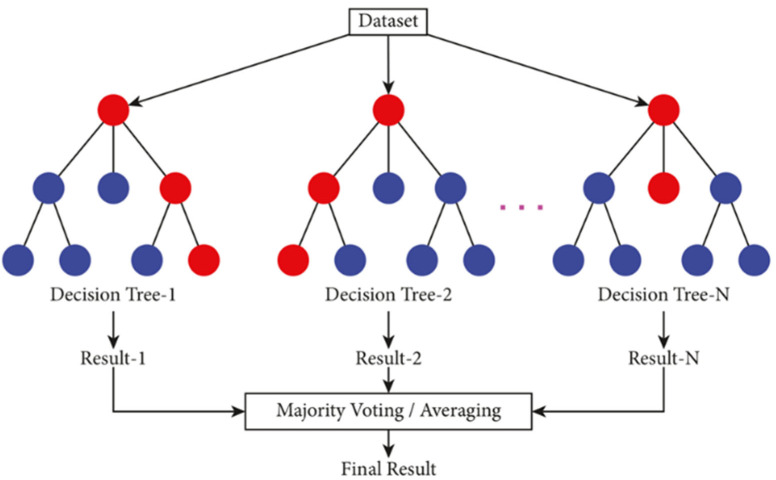
Example of the Random Forest algorithm [14]. Red circles represent decision nodes (internal nodes where a splitting decision is made based on a feature). Blue circles represent leaf nodes (terminal nodes that output the final prediction for a tree, such as a class label or regression value). Each Decision Tree (1 to N) is trained on a random subset of data and features. The leaf nodes produce individual results, which are combined via majority voting (for classification) or averaging (for regression) to produce the Final Result.

Machine learning can address the matter of complexity in this scenario. In this example, a model called Random Forest regression can be used to estimate scoliosis progression risk. As the name suggests, a Random Forest regression consists of several individual regression trees like the one depicted in Figure 2.

The magnitude of progression for the entire Random Forest is obtained as a combination of the results from the individual trees. These individual trees are visually easy to understand and automatically take interactions into account due to the cascading nature. No user-based model choice needs to be performed beforehand, as all interaction terms are data-driven. This allows the individual trees and the resulting Random Forest to effectively manage a large number of predictors. Although the interpretability of an entire forest is difficult relative to the individual trees, the ability to predict progression magnitude is greatly improved owing to the high accuracy of the model based on the complex interactions between variables.

**Figure 3:**
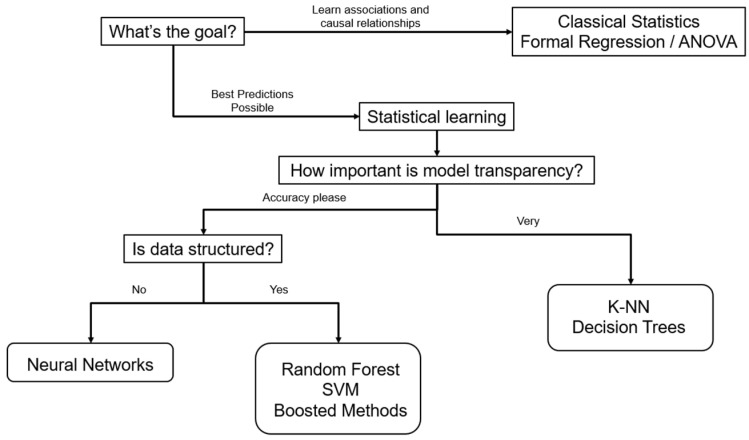
Off-the-Shelf Machine-Learning Algorithms for Tabular Data. ANOVA = Analysis of Variance, SVM = Support Vector Machine, K-NN = K-Nearest Neighbors.

Recent studies have applied Random Forest regression algorithms to predict scoliosis progression [15,16] as well as to predict patients’ Spinal Injury Association Impairment Scale in patients with spinal cord injury [17]. The Spinal Cord Injury (SCI) Models Systems database is a multicenter prospective database of patients with SCI, but a large percentage of data was lacking when it came to the American Spinal Injury Association (ASIA) impairment scales of these patients on admission. Five ML algorithms were trained using a large subset of patients with complete ASIA impairment scales, and the best-performing algorithm, called Random Forest, was shown to have 81.7% accuracy, 76.3% sensitivity, and 93.8% specificity when validated against linear regression models. Figure 3 illustrates the authors’ preferred algorithm for choosing the proper off-the-shelf ML methods based on study data and purposes.

## 3. Machine-Learning (ML) Applications in Pediatric Spine Care

ML models can enhance pediatric spine care by predicting surgical outcomes, curve progression, and clustering patients. To date, this is still mostly constrained by small, single-center datasets and the absence of external validation. The training of ML models can be categorized into supervised or unsupervised learning depending on the availability of ground truth labels [18]. Supervised models like Random Forests show promise in forecasting brace success, while unsupervised clustering discovers patterns in data without using labels [15,19].

**Figure 4:**
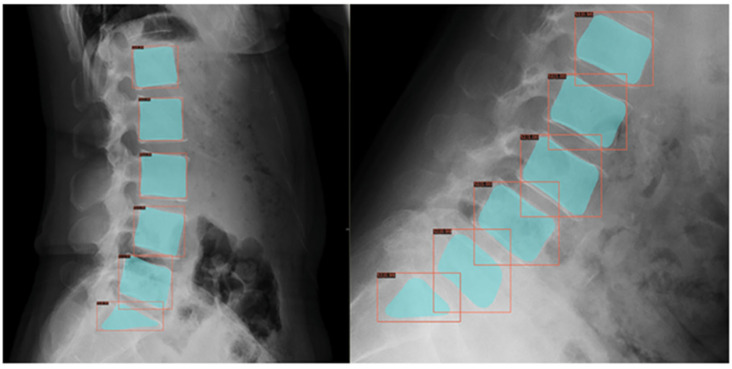
Schematic diagram of lumbar spine X-ray results after an instance segmentation model for detecting spondylolisthesis [20].

As unsupervised learning identifies patterns without labels, it is useful for patient clustering. A single-center study applied semi-supervised clustering to 111 adolescent sagittal spine radiographs, identifying three to five distinct alignment groups, with sagittal vertical axis found as a key differentiator to classify scoliosis subtypes or treatment responses. This still lacked external validation and generalizability assessment [19]. Lumbar spondylolisthesis diagnosis in adolescents can also be automated using ML (Figure 4), although primarily studied in adults [20].

**Figure 5:**
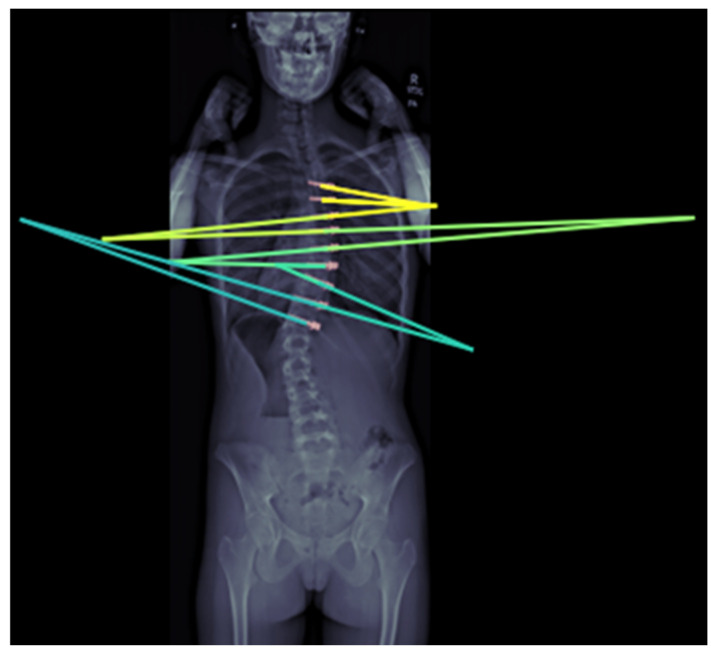
Validated inter-screw angle measurement model using DL algorithm.

ML models are able to predict surgical outcomes such as length of stay (LOS), estimated blood loss (EBL), and operative time. Using 6076 cases from a multicenter registry, a study developed a risk-stratified benchmarking tool for AIS surgeries. Although predicting quality-of-life scores was less successful, ensemble ML methods outperformed traditional regression for LOS (up to 64% accuracy within 1 day when trained on the last 5 years), EBL (up to 83.05% accuracy within 326 mL of the mean EBL when trained on the last 5 years), and operative time (up to 84% accuracy within 111 min when trained on the entire time frame of the study). The key limitation, however, is the inability of the models to predict health-related quality of life scores (SRS-Pain and SRS-Self-Image) in a useful manner, which highlights the challenge of applying machine learning to these more subjective, patient-reported outcomes [21]. To monitor possible vertebral body tether (VBT) breakage, a DL algorithm for automating inter-screw angle measurements in VBT has been developed and validated to be accurate to within 0.66° on average [4] (Figure 5).

**Figure 6:**
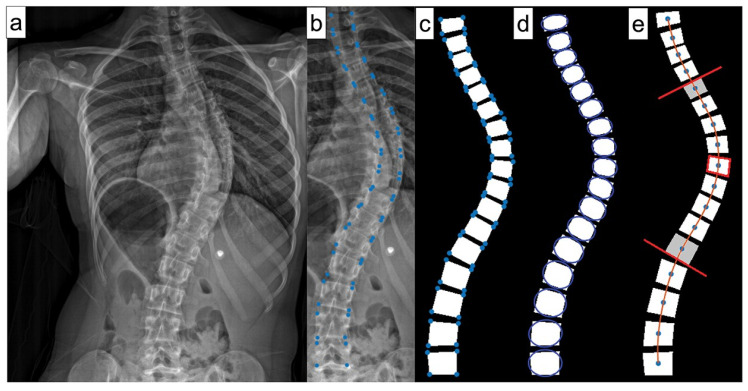
Steps of Cobb angle measurement for AI training from frontal radiographs. (**a**) Plain frontal X-ray. (**b**) Four landmark points (LMPs) were selected per vertebra. (**c**). Polygon fit through the LMPs of each vertebral vertex. (**d**) Best fit ellipse through each polygon to calculate the orientation of each vertebra as the angle between the major axis of the ellipse and the horizontal. (**e**) A cubic spline was fit through the centroids of the vertebrae, the most tilted vertebrae above and below the apical level were identified, and the Cobb angle was calculated between these vertebrae [15].

ML can also effectively forecast spinal curve progression, aiding in timely intervention decisions. Using features like initial Cobb angle (Figure 6) and Risser stage, a Random Forest model has been shown to be able to predict final Cobb angles in 193 AIS patients from a single center with a 4.64° mean absolute error [15]. Multiple models have also been developed to help identify screw brands, which is helpful for preoperative planning in revision spine surgeries [22,23,24]. Another model is currently being developed to differentiate scoliosis etiology from plain radiographs alone. It has shown superior performance compared to two senior spine surgeons [25].

**Figure 7:**
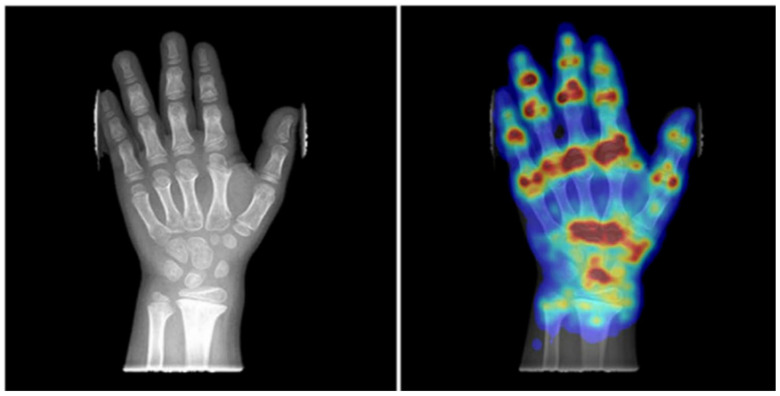
Radiograph and corresponding attention map produced by the hand bone age DL model for a 7-year-old girl with achondroplasia [26].

Hand bone age plays a crucial role in managing pediatric spinal deformities; however, accurately measuring it can be challenging in patients with skeletal dysplasias, as these conditions often result in significant hand bone malformations. Recently, an open-source prior-free DL model has been specifically developed and validated to mitigate this problem in patients with achondroplasia, hypochondroplasia, intrauterine growth restriction, Noonan syndrome, pseudohypoparathyroidism, SHOX gene mutation, Silver–Russell syndrome, Ullrich–Turner syndrome, and other skeletal dysplasias without genetic abnormalities. (Figure 7) Despite limited validation to specific syndromes and small cohorts, it has been found to be effective in evaluating age and tracking the development of both normal and dysplastic bones, and comparable with clinical ground truth assessment [26].

**Figure 8:**
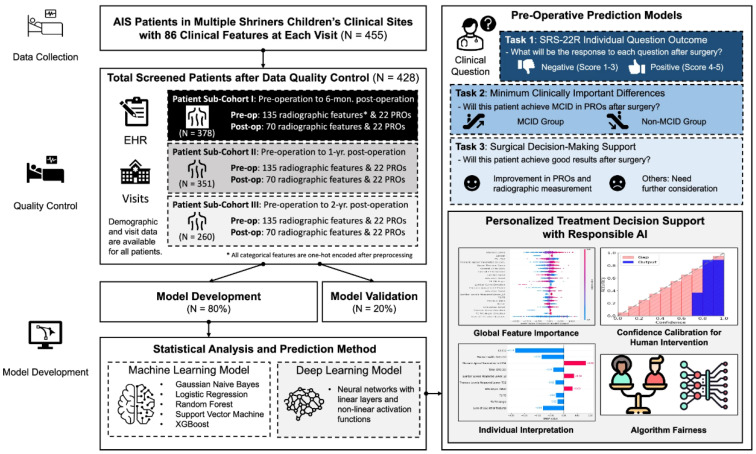
Overview of an AI-driven support system for personalized surgical planning and outcome prediction in AIS patient rehabilitation following PSF surgery from a multicenter pediatric patient cohort.

Another group of researchers has successfully developed an AI-enabled surgical planning and counseling support system to predict postoperative outcomes in AIS patients undergoing posterior spinal fusion (PSF) surgery, using a rare multi-site cohort of 455 patients from Shriners Children’s hospitals [27]. The model predicts three key outcomes to support shared decision-making: individual responses to the refined Scoliosis Research Society-22 (SRS-22R) questionnaire, likelihood of achieving clinically meaningful improvements, and changes in both radiographic measurements and patient-reported outcomes (PROs). To the authors’ knowledge, it is the first known model to predict SRS-22R responses post-PSF, leveraging follow-up data spanning 6 months to 2 years. The framework integrates explainable AI techniques to identify important predictive features, calibrates model confidence for appropriate human oversight, and addresses gender bias (Figure 8).

Finite element (FE) analysis is a physics-based computational method used to simulate and solve complex engineering problems, such as stress, heat transfer, and fluid flow, by breaking down the systems into smaller, manageable elements. In contrast, DL models use neural networks to learn direct input–output mappings from data (e.g., preoperative imaging to postoperative alignment) without explicit physical constraints, enabling faster inference but potentially reduced interpretability and generalizability outside training distributions. FE analysis relies on deterministic models rooted in classical physics, making it particularly suited for scenarios where the main equations are well-understood and precision is critical. A recent study introduced both experimental and in silico model-based evaluation methods to assess the corrective effect of a newly developed hybrid scoliosis brace, based on initial performance testing and the construction of a patient-specific finite element model [28]. Using FE simulations on 64 patients, another study showed it was able to predict postoperative spinal shapes in real time, achieving a 3.75 mm position error [29]. A multicenter study recently validated a patient-specific finite element model combined with a growth modulation algorithm to accurately predict two-year outcomes of lumbar VBT, demonstrating its potential to enhance surgical planning and improve treatment consistency in idiopathic scoliosis [30]. Both are mechanistically grounded but computationally intensive and not yet prospectively validated.

Overall, current challenges include small datasets, limited validation, and clinical integration. A systematic review of 63 studies confirmed ML use in diagnosis (n = 38), outcomes (n = 11), prognosis (n = 7), and risk (n = 7), but > 80% had n < 300, single-center designs, and no external validation, which highlights a critical evidence gap [31]. Future research should prioritize multicenter studies and real-time ML tools.

## 4. Deep Reasoning and Learning for Volumetric Analysis

Deep reasoning and learning (DR) refers to an AI system that integrates a perception module, typically a neural network for feature extraction from raw data (e.g., CT scans), with a reasoning module that performs multi-step logical analysis, often using symbolic or probabilistic frameworks to emulate human-like problem-solving. Unlike standard DL, which relies on end-to-end neural networks optimized for pattern recognition with large annotated datasets, DR combines data-driven learning with structured reasoning to generalize from fewer examples. DR emphasizes flexible, probabilistic reasoning over rigid rule-based systems, enabling better handling of complex, dynamic data like scoliosis imaging. DR’s advantage lies in its reduced need for annotated data due to the reasoning module’s ability to infer relationships from limited examples, unlike DL’s dependence on extensive labeled datasets [32]. Recently, a new DR-based model has been developed by researchers at Cornell to automate the volumetric assessment of Hounsfield Units (HU), trained on a combination of publicly available CT datasets and deidentified institutional lumbar CT scans, and our team has internally validated the model against DXA (Dual-energy X-ray Absorptiometry) metrics [33]. The volumetric HU correlated most strongly with DXA bone mineral density (BMD) at L2 (Spearman ρ = 0.75, *p* < 0.0001). The model was able to efficiently segment each vertebra and quantify both the volume and volumetric HU of the whole imaging studies from all available kernels in about 30 s per CT scan.

**Figure 9:**
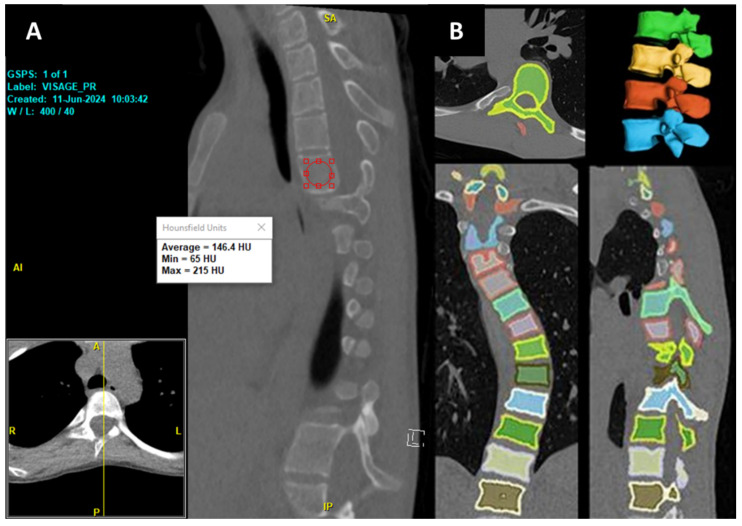
(**A**) Conventional mid-sagittal method of opportunistic HU measurement involves selecting a single mid-sagittal slice of a vertebral body and placing an elliptical region of interest (ROI) within the trabecular bone to measure the HU. (**B**) Automated vertebral segmentation generated from the DR-based model.

In addition to the DXA, we found that the same DR model also correlated strongly with surgeons’ intraoperative assessment of the operated vertebrae [34]. Additionally, it has also been able to assess the volume and volumetric HU for neurofibromatosis type 1 patients who underwent spine surgery, showing significantly lower bone density than normal controls [35]. This provides a faster, more objective, and comprehensive alternative to traditional manual methods. This is especially true since the conventional mid-sagittal method of opportunistic HU measurement is confounded by a constant shift in the sagittal plane due to the scoliotic condition of the vertebrae (Figure 9), as well as variability in HU values at the vertebral bodies. However, these findings are based on small, single-center cohorts, limiting generalizability. Validation in larger, multicenter cohorts is essential to confirm the model’s reliability, accuracy, and clinical utility for preoperative planning and spinal bone health monitoring, particularly in diverse scoliotic populations.

## 5. Large Language Models (LLM) in Practice

LLMs are State-of-the-Art AI algorithms that can understand input texts and generate responses in human languages. Compared with traditional natural language processing (NLP) algorithms, which are usually developed for performing a limited set of tasks, LLMs are trained to grasp a broader knowledge base and to be smart enough to learn new tasks or knowledge from instructions provided in the input prompt. These advanced and versatile AI techniques have been studied and evaluated for different healthcare applications, such as making diagnoses, information extraction, summarization, and generating medical reports [36]. Commercial models such as GPT-4 have been evaluated and demonstrated to be able to generate accurate, safe, and helpful neurosurgical information [37]. Therefore, LLMs hold significant promise in streamlining and enhancing the workflow for various pediatric spine care [38,39,40]. In the future, these may include a surgeon performance program from the Setting Scoliosis Straight Foundation or registry participation in Harms, or the Pediatric Spine Study Group. Currently, the study group requires surgeons to complete three distinct forms: Pre-Operative and Operative, Peri-Operative and Early Post-Operative, and Complications Within 90 Days of Surgery, with plans to expand to 1- and 2-year follow-up timepoints. Data abstraction can be time-consuming and prone to inconsistency.

Recent studies have shown that LLMs can be employed to automatically extract structured data from unstructured EHR [41,42]. Although requiring further study for validation, these results are promising as those LLMs may also be used to analyze spine surgery-related EHR, including surgical history, intraoperative details, and postoperative outcomes. This capability could drastically reduce manual data entry, improve data quality, and ensure that critical information is captured uniformly across cases. To ensure reliability, human-in-the-loop review is essential, where clinicians verify LLM outputs to catch errors or hallucinations, supported by audit trails that log model decisions and human interventions for traceability and accountability.

In addition to automating form completion, LLMs can also support physicians by summarizing medical reports and generating concise, structured reports for case conferences or team discussions [43]. Once adapted specifically to pediatric spine care, these AI-generated summaries can highlight relevant metrics such as blood loss, instrumentation used, and any intraoperative complications, making it easier to track individual performance trends over time. Furthermore, LLMs can function as AI scribes during clinic documentation [44], supporting clinical decision-making through real-time access to guidelines and patient-specific insights. To mitigate risks of inappropriate outputs, prompt and content filtering techniques can restrict LLMs to generate only clinically relevant responses, while domain-constrained retrieval corpora, e.g., tailored to pediatric spine care, enhance accuracy by limiting knowledge sources to verified medical literature.

Currently, there are about 90 commercially available software programs utilizing LLMs to enhance provider efficiency and strengthen payer–provider alignment by automating the prior authorization process, thereby reducing the manual workload for clinicians and staff [45]. However, it is important to also note the limitations and concerns of LLMs. First, LLMs may not be trained to obtain specialized knowledge during their development/training. This may lead to LLM hallucinations, where the models generate untruthful, misleading, and hence harmful content for clinical use. Techniques, including retrieval-augmented generation (RAG) to provide LLMs with the right context or knowledge, can mitigate this challenge [46]. Second, as clinical documents contain protected health information (PHI) in various forms, it is important to fully deidentify them before sending them to proprietary LLMs, e.g., ChatGPT (https://chatgpt.com/, accessed on 10 October 2025). For enhanced PHI protection, local or on-premises LLM deployments can minimize data exposure risks, ensuring compliance with regulations like the Health Insurance Portability and Accountability Act (HIPAA) while maintaining control over sensitive pediatric spine care data.

## 6. AI in the Operating Room (Or)

Aside from offering significant opportunities with regard to patient selection, outcome prediction, and surgical planning, AI also has the potential to support intraoperative decision-making, particularly in cases where preoperative data alone cannot capture the full clinical complexity or when intraoperative conditions change, necessitating advanced surgical skills. While the AO standard trajectory for pedicle screw placement is widely used, it may not provide optimal pullout force in patients with low BMD, such as neuromuscular scoliosis [47] and patients with neurofibromatosis type 1 (NF-1) [48]. Recent novel AI software has shown promise in optimizing screw trajectories to target higher BMD regions, thereby enhancing screw fixation [49]. Notably, AI-based planning has demonstrated improved pullout force in patients with BMD between 40 and 120 mg/cm^3^, though its effectiveness is limited in patients with very low BMD (0–40 mg/cm^3^), where alternative strategies such as bicortical or cement-augmented screws may be more beneficial. In a clinical cohort of 50 AIS patients, robotic pedicle screw placement using a commercial system significantly reduced breach rate and provided better trunk shift and radiographic shoulder height correction, with preserved lumbar lordosis [50].

**Figure 10:**
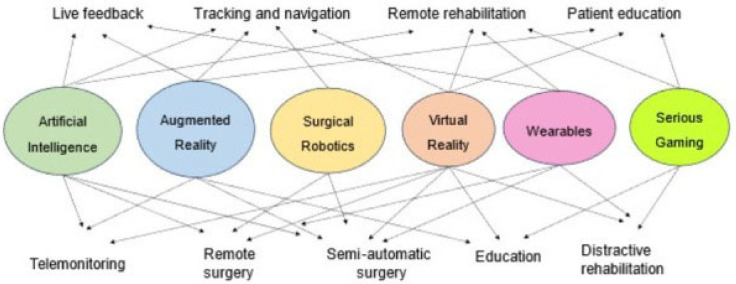
Potential integrations and applications of AI, VR, and AR [52].

Surgical simulation is an increasingly valuable educational tool, offering trainees the opportunity to practice in a safe, standardized, and controlled environment. A recent study highlights the strong educational potential of Virtual Reality (VR) in spine surgery training, particularly for early-stage trainees [51]. Results showed notable improvement in anatomical knowledge, especially among junior trainees, with an 11.4% average increase in post-test scores. When combined with immersive technologies, such as Augmented Reality (AR) and VR, AI can even further elevate surgical education in pediatric spine care. The synergistic integration of AI, VR, and AR into emerging healthcare technologies holds immense potential to transform how surgeons learn, plan, and operate [52]. (Figure 10) Future studies could explore more into the potential of their integration.

## 7. Ethical, Practical, and Limitations of ML Models

Guidelines for the ethical use of AI in medical research are currently being updated and addressed. Some of these guidelines apply to research within subspecialties, including orthopedics. In general, these advocate for transparency of the source and type of data to be analyzed, in addition to ensuring its accuracy. Any data that is excluded should be described with justifiable reasoning. The model, algorithm, framework, and architecture of the AI used should be defined, as well as the process to train it. Any parameter that influences how the AI responds to the data should also be described. To explain the model’s performance, results should be contextualized by the techniques used to achieve them, and the researcher should try to hypothesize the existence of any output outliers [53].

Some concern has been raised that the adoption of AI can put protected health information (PHI) at risk of disclosure to outside parties. Even if traditional PHI parameters such as name, address, and date of birth are carefully protected, multimodal data allow for re-identification of even anonymized datasets. Deidentified PHI analyzed by DL models can be matched to information scraped from patient portals via user tracking code. Recently, Facebook and several healthcare companies were named in a lawsuit for allegedly allowing the tracking code Meta Pixel to access patient information within healthcare portals [54]. As AI continues to evolve in its use for academic research, investigators must update and adapt the processes necessary to rein in threats to patient privacy, limiting the scope of AI to prevent any breaches of confidentiality. For instance, LLMs must live within the institutional firewalls in order to prevent PHI and patient data from being transmitted to commercial websites and potentially disclosed to outside parties. European privacy laws have more stringent protections against the release of individual information.

Another primary ethical consideration is that bias within data that DL is trained on may create biased models and further contribute to unequal outcomes for diverse and/or underrepresented patient populations. Although children represent 30% of the global population, they are largely underrepresented in the datasets that fuel advancements in AI for medical imaging, accounting for less than 1% of publicly available medical imaging datasets. This may yield to age-biased models with systematically higher false positives in younger patients (e.g., adult-trained chest X-ray classifiers misdiagnose cardiomegaly more often in infants) [55]. Recent studies show that appropriate data preprocessing, using pre-established safeguards, and using optimal hyperparameters can help mitigate model bias. Balanced datasets would help diminish this problem before it starts, but these are difficult to create because of longstanding problems with unbiased recruitment. Using extensive datasets from large-scale cohorts and multiple sources is necessary and has been shown to improve the generalizability of AI models [56].

Prognostication for the orthopedic surgeon can be a challenging task, considering the complexity of variables including patient demographics, extent of injury on imaging, patient history, and comorbidities. This area is ripe for the development of AI models that can sort through the interplay of these variables and the use of digital twins and prior matched experience and patient characteristics to predict future outcomes. Future semi-autonomous robotic platforms could have complex AI inputs as spine surgery becomes increasingly automated. Training these DL models carefully to avoid bias and prepare for anatomic and patient contingencies is necessary. Within the confines of privacy limitations, ensuring transparency of the data used for training and testing is necessary, as well as carefully hypothesizing what happens in the AI “black-box”. Spine surgery is challenged by the lack of publicly available training datasets, especially for patients with specialized disease states such as scoliosis. Ensuring patient privacy throughout the process should be at the forefront of the investigators’ concerns as scientific advancement progresses, especially when treating pediatric patients.

While small sample sizes and growth variability pose validation challenges [8,9,10], pediatric applications introduce distinct ethical issues, including assent (vs. consent) in minors and long-term AI monitoring during growth. For rare deformities such as congenital scoliosis, AI holds promise but faces regulatory hurdles, such as FDA pediatric-specific clearance requirements and low patient volumes, which limit the financial viability of pediatric device development. Along with each institution’s unique requirements, investigators must also prioritize assent protocols, longitudinal privacy safeguards, and compliance with the FDA Pediatric Research Equity Act (PREA) to protect this vulnerable population.

To ensure ethical and effective AI deployment in pediatric spine research, a structured framework may be made as follows: (1) Identify time-intensive workflows as research question (e.g., manual Cobb angle measurement, literature synthesis, or outcome annotation) to target high-impact automation; (2) Find established, HIPAA-compliant tools from trusted vendors with transparent data policies, avoiding unproven startups for PHI; (3) Verify security and compliance via institutional firewall integration and compliance with other requirements such as HIPAA Business Associate Agreement (BAA); (4) Pilot on noncritical tasks (e.g., deidentified public datasets or administrative summarization) to assess reliability without patient risk; (5) Critically evaluate impact using pre/post metrics (e.g., time saved, error rates, researcher satisfaction); and (6) Review and iterate to track tool updates and possible bias reports, as well as regulatory changes.

Clinicians and researchers should be cognizant of the inherent technical limitations of ML models. ML often involves a large number of model hyperparameters, and the choice of these parameters can significantly impact the performance of an ML method. Without a clear description of how these parameters are selected, it is challenging to reproduce results in an ML application, even when the same data are used. In many existing studies, when ML is used, these details are not sufficiently clear.

Additionally, ML methods are often complex and involve a large number of parameters, making overfitting likely. When overfitting happens, an ML method may fit the training data well but predict new data poorly, especially when the sample size is limited and the number of features is large. This risk should always be considered, and techniques such as cross-validation, regularization, feature selection, and ensuring sufficient sample size are essential to prevent overfitting.

## 8. Conclusions

AI offers significant potential to revolutionize pediatric spine care, with capabilities ranging from image sorting and surgical outcome prediction to curve progression forecasting, volumetric analysis, and intraoperative decision-making. LLMs further enhance their utility in clinical settings and strengthen payer–provider alignment. Despite these advancements, hurdles such as unclear implementation guidelines, model opacity, ethical concerns, and data limitations remain substantial barriers to widespread adoption. Moreover, risks such as AI hallucinations in LLMs (e.g., fabricating progression trajectories) or overfitting in ML models (e.g., poor generalization from small pediatric cohorts) must not be understated. Addressing these challenges requires a concerted effort through multicenter studies, real-time AI tools, and interdisciplinary collaboration among clinicians, researchers, and AI specialists. This must be complemented by rigorous prospective studies, external validation, and ongoing monitoring to effectively mitigate potential risks. Such efforts are essential to ensure AI’s integration into pediatric spine care is safe, effective, and accessible, with great potential to enhance patient outcomes and improve healthcare delivery.

## Data Availability

No new data were created or analyzed in this study.
